# Performance monitoring and the medial prefrontal cortex: a review of individual differences and context effects as a window on self-regulation

**DOI:** 10.3389/fnhum.2012.00197

**Published:** 2012-07-11

**Authors:** Stefon J. R. van Noordt, Sidney J. Segalowitz

**Affiliations:** Cognitive and Affective Neuroscience Laboratory, Department of Psychology, Brock UniversityON, Canada

**Keywords:** ERN, FRN, Nogo N2, anterior cingulate, medial prefrontal cortex, individual differences, performance monitoring, self-regulation

## Abstract

The medial prefrontal cortex (MPFC) is central to self-regulation and has been implicated in generating a cluster of event-related potential components, collectively referred to as medial frontal negativities (MFNs). These MFNs are elicited while individuals monitor behavioral and environmental consequences, and include the error-related negativity, Nogo N2, and the feedback-related negativity. A growing cognitive and affective neuroscience literature indicates that the activation of the anterior cingulate cortex (ACC) and surrounding medial prefrontal regions during performance monitoring is not only influenced by task context, but that these patterns of activity also vary as a function of individual differences (e.g., personality, temperament, clinical and non-clinical symptomatology, socio-political orientation, and genetic polymorphisms), as well as interactions between individual differences and task context. In this review we survey the neuroscience literature on the relations between performance monitoring, personality, task context, and brain functioning with a focus on the MPFC. We relate these issues to the role of affect in the paradigms used to elicit performance-monitoring neural responses and highlight some of the theoretical and clinical implications of this research. We conclude with a discussion of the complexity of these issues and how some of the basic assumptions required for their interpretation may be clarified with future research.

A hallmark of self-regulation is flexibility—the ability to maintain or disengage and establish different patterns of behavior in pursuit of adaptive outcomes (Baumeister et al., 2007). Monitoring, detecting, and evaluating behavioral and environmental consequences require the coordination of activity across multiple neural systems. In the human brain, areas of the prefrontal cortex are involved in mediating cognitive control processes of motor behavior (Ridderinkhof et al., 2004a,b; Polli et al., 2005), as well as appraisal and motivational responses to behavioral and environmental feedback (Ridderinkhof et al., 2004b; Schnider et al., 2005; Diekhof et al., 2011; Etkin et al., 2011). It is well established that activation of the anterior cingulate cortex (ACC) and surrounding medial prefrontal areas is associated with performance monitoring processes such as error detection and response correction, stimulus-response conflict resolution, inhibitory control, and feedback evaluation, all of which involve demands on the selection and maintenance of goal-directed behavior (Holroyd and Yeung, 2012). In addition, accumulating evidence is showing that the activation in the ACC and medial prefrontal cortex (MPFC) during performance monitoring is not only influenced by task context, but that these patterns of activity also vary as a function of individual differences in personality, as well as interactions between personality and task context.^1^

There is considerable interest in trying to understand the associations among personality, context, and brain activation during performance monitoring, as reflected by a growing body of literature in the cognitive, affective, and social neurosciences. Not only does this research have important theoretical implications, but these data can also inform our clinical understanding about how neurophysiological differences may reflect pathological patterns of performance monitoring and self-regulation. In order to synthesize the current understanding in the field, we have surveyed for this paper the neuroscience literature on the relations between performance monitoring, personality, task context, and brain functioning with a focus on the MPFC. The growth in research on the functional relations between MPFC and these issues has been exponential in the last decade, as indicated by a literature search in Google Scholar (see Figures 1A and B). In this review, we relate these issues especially to the role of affect in the paradigms used to elicit the performance monitoring neural responses. To simplify the terminology, we will consider the DMPFC and VMPFC as a broad division, with each of these areas including several anatomically distinct regions (see Figure 2).

**Figure 1:**
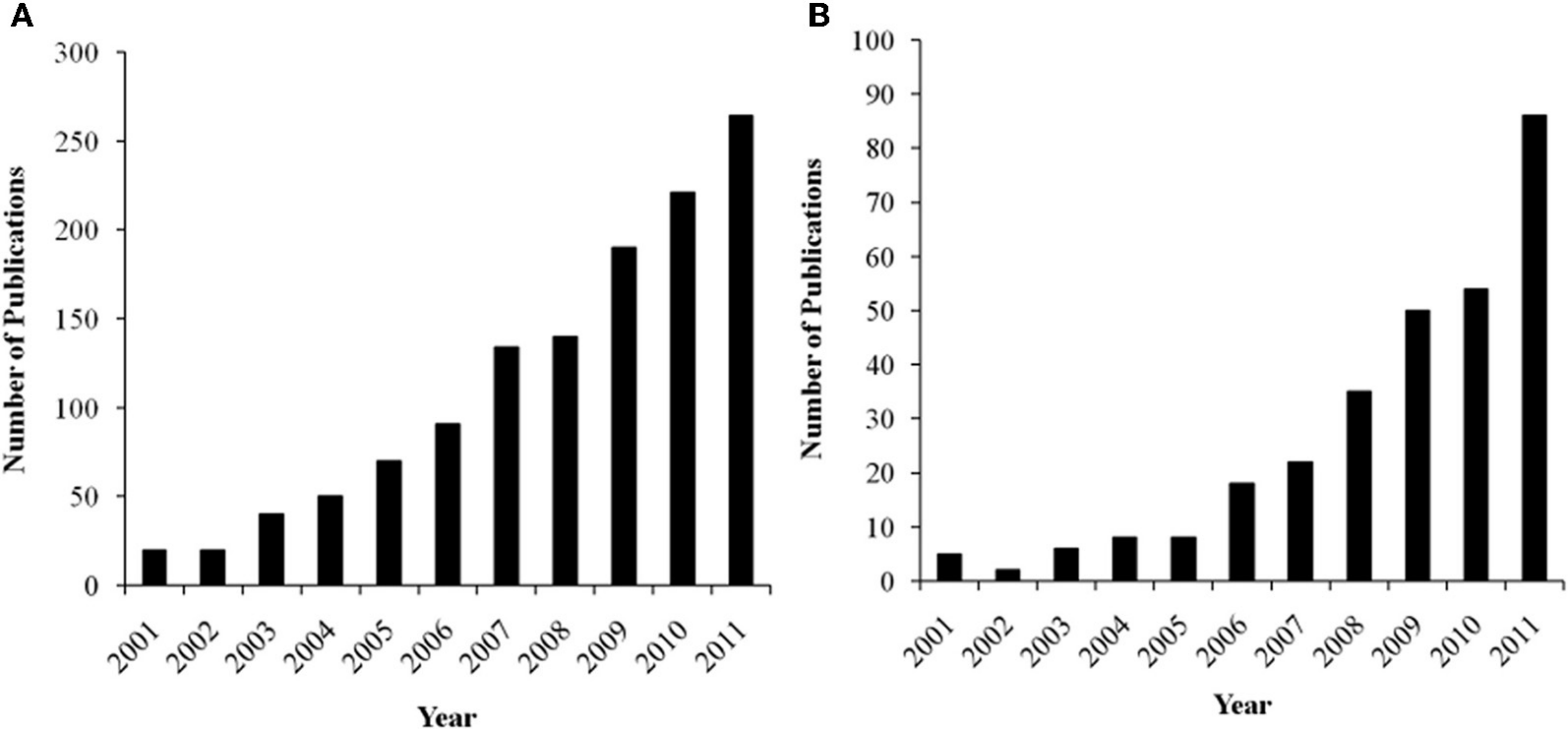
**(A)** Number of annual publications listed in Google Scholar for key-terms “error-related negativity + personality.” **(B)** Number of annual publications listed in Google Scholar for key-terms “feedback-related negativity + personality.”

**Figure 2:**
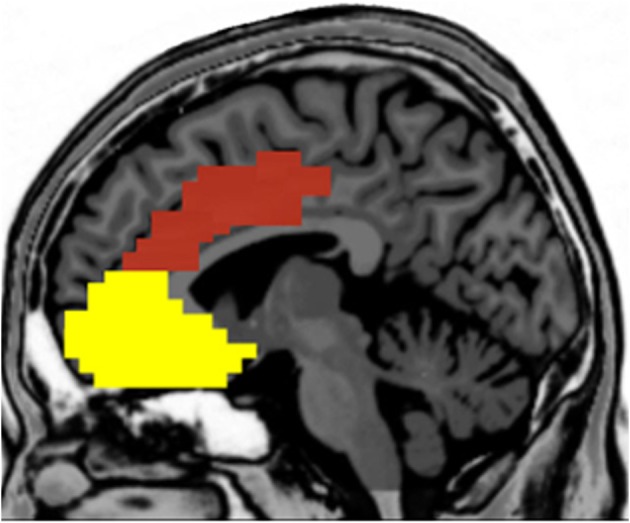
**Broad division between the dorsal (red) and ventral (yellow) medial prefrontal cortices**.

## The medial prefrontal cortex and medial frontal negativities

**Figure 3:**
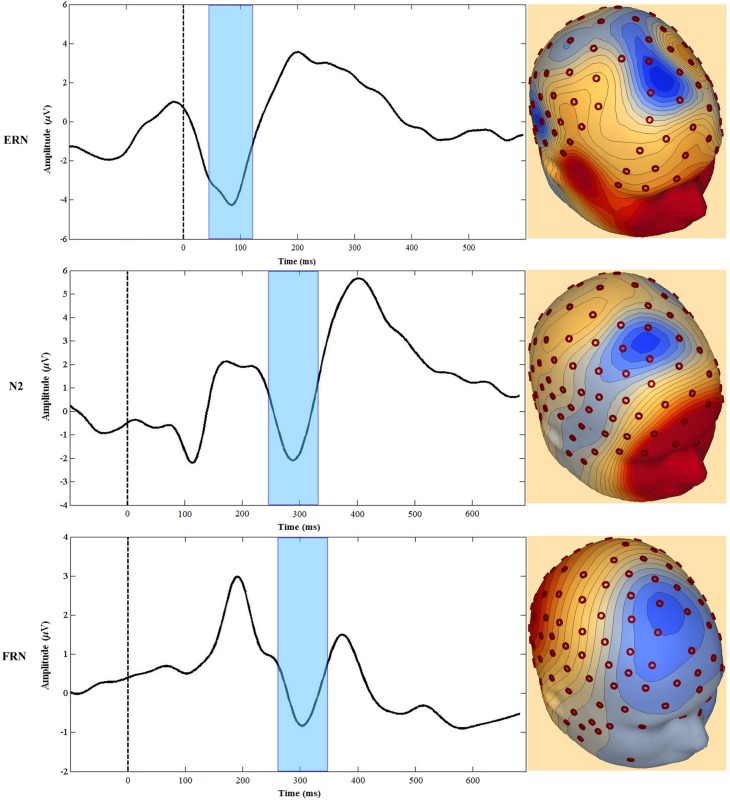
**Examples of waveforms and topographic voltage maps illustrating similarities between the ERN, Nogo N2 (N2), and feedback-related negativity (FRN).** The FRN waveform is the difference between losing and winning trials in a gambling task (see text). The dashed line at 0 ms represents the onset of the time-locked event, and the shaded area highlights the peak negativity.

The MPFC generates several event-related potential (ERP) components associated with performance monitoring and self-regulation. For our focus, these ERP components include the error-related negativity (ERN), the Nogo N200 (N2), and the feedback-related negativity (FRN). Although some researchers have used the term medial frontal negativity (MFN) to describe specifically the FRN (e.g., Gehring and Willoughby, 2002), for this review we use the MFN label when referring to all three components. Each of these MFNs is elicited in a specific context (Luck, 2005) and, as described below, regions of the MPFC are consistently implicated as neuronal generators of all three (Gehring and Willoughby, 2002; van Veen and Carter, 2002a,b; Mathalon et al., 2003; Amodio et al., 2007; Gentsch et al., 2009; Segalowitz et al., 2010). Furthermore, there are theoretical constructs linking the three and therefore, while we will not at all claim that they are identical, there are good reasons to consider the three components together (see Figure 3).

### ERN

The ERN, or error-negativity (Ne; Falkenstein et al., 1991), was first identified in the early 1990s and was thought to reflect the activation of a neural system sensitive to discrepancies between intended and actual responses. This ERP component can be observed as a negative-going deflection over central and frontal midline sites, peaking between 50 and 100 ms after an erroneous response has been delivered (Gehring et al., 1993). The ERN is traditionally examined using speeded response tasks in which conflicting stimulus-response mappings are equally likely to occur, such as in a stimulus discrimination task with incongruent flanking stimuli, or when prepotent responses to target stimuli must be inhibited, as is the case in a Go/Nogo task. The elicitation of the ERN is not specific to errors committed with the hand, as it has been observed after foot (Holroyd et al., 1998), vocal (Masaki et al., 2001) and saccadic motor errors (Van ‘t Ent and Apkarian, 1999; Murphy et al., 2006), and indeed even when making partial mistakes (Vidal et al., 2000; Masaki and Segalowitz, 2004) or only observing errors made by others (Miltner et al., 2004). Based on data from lesion, functional neuroimaging, and electroencephalography (EEG) source modeling methods, error-related responses have been localized to the dorsomedial prefrontal cortex (DMPFC) (Gehring et al., 1993; Dehaene et al., 1994; Carter et al., 2000; van Veen et al., 2001; van Veen and Carter, 2002a; Luu et al., 2003; Herrmann et al., 2004; Milham and Banich, 2005; Amodio et al., 2007; O'Connell et al., 2007) and, in some studies, ventromedial regions (Kiehl et al., 2000; Menon et al., 2001; Luu and Tucker, 2003; Stemmer et al., 2003; Ridderinkhof et al., 2004a; Swick and Turken, 2004; Taylor et al., 2007). Convergence has been observed across different functional measures, such that error-related scalp potentials correlate with functional magnetic resonance imaging (fMRI) signals (Mathalon et al., 2003) and current source density (CSD; van Noordt, 2012) in the MPFC.

### Nogo N2

When the participant has to withhold a response in the midst of responses that have become habitual (or prepotent), the N2 component of the ERP normally has an increased amplitude (Nieuwenhuis et al., 2003)^2^. Whereas the ERN is time-locked to response onset, the N2 is locked to the stimulus signaling that a response is to be withheld. The N2 has a similar scalp distribution as the ERN, peaking maximally over central and frontal midline sites and, using source analysis, has been shown to share neural generators in the medial frontal cortex (Bokura et al., 2001; Ullsperger and von Cramon, 2001; van Veen and Carter, 2002a,b; Bekker et al., 2005; Jonkman et al., 2007; Amodio et al., 2008; Gründler et al., 2009). As with the ERN, fMRI activation in the MPFC during inhibition of a response to a Nogo cue has also been shown to relate to N2 scalp amplitudes (Mathalon et al., 2003).

### FRN

The FRN is similar to the N2 in that it is a stimulus-locked component, is negative in polarity, peaks at a similar latency (approximately 250 ms post-feedback) and therefore may be considered part of the N2 family (see Holroyd, 2004, for a discussion). Compared to the ERN, which reflects the activation of an internal monitoring system, the FRN reflects activity associated with external monitoring (Gentsch et al., 2009) and is time-locked to the external feedback stimulus informing the participant about an environmental (e.g., win or loss) or behavioral (e.g., correct or incorrect) consequence. Typically, the FRN is investigated using gambling (Gehring and Willoughby, 2002) or associative learning paradigms (Nieuwenhuis et al., 2004) in which individuals make choices between stimuli that are characterized by varying features (e.g., riskiness, magnitude, and probability), or attempt to learn action-outcome contingencies on the basis of feedback information (Holroyd and Coles, 2008). The FRN component is not modality specific (Miltner et al., 1997), and considerable evidence suggests that the FRN reflects, to some extent, the evaluation or appraisal of outcomes (Luu et al., 2003; Holroyd et al., 2006), particularly in the context of reinforcement learning (Yeung et al., 2005; Holroyd et al., 2009; Pfabigan et al., 2011). Several groups have reported larger FRN amplitudes to feedback indicating that behavior was incorrect (Miltner et al., 1997; Sato et al., 2005) or that an outcome has resulted in a loss or punishment (e.g., Gehring and Willoughby, 2002; Pfabigan et al., 2011). In addition, FRN amplitude is sensitive to prediction errors (Holroyd and Coles, 2002), unexpected outcome deviations (e.g., false-positive feedback; Oliveira et al., 2007), and predicts future behavioral responses, such as the avoidance of choices which were previously incorrect (Yasuda et al., 2004; Cohen and Ranganath, 2007; van der Helden et al., 2010), or the acceptance of unfair offers from others (Hewig et al., 2011).

Scalp distributions for the FRN suggest that peak activation occurs over sites slightly more anterior to those at which the ERN and N2 are often found to be maximal (Gehring and Willoughby, 2004; Muller et al., 2005; Luu et al., 2009), and are accounted for by source models which often include VMPFC regions (Luu and Posner, 2003; Muller et al., 2005; Nieuwenhuis et al., 2005; Luu et al., 2007; Kamarajan et al., 2009; Polezzi et al., 2010; Segalowitz et al., 2010), suggesting the possibility of additional underlying cortical generators. Nevertheless, similar regions of the perigenual ACC implicated in generating the ERN have been found for the FRN (Bellebaum and Daum, 2008), and results from our lab suggest that activation in both ventral and dorsal medial regions of the PFC at the time of error feedback correlate with FRN scalp amplitudes (Segalowitz et al., 2010, 2012; van Noordt, 2012).

Although not the focus of the present review, it is worth noting that relatively little research has considered the relative regional contribution from areas of the MPFC (e.g., dorsal-ventral) in generating these ERPs, and whether this may vary due to contextual influences because of the impact on arousal and affect. By extension, not much research has focused on how the interactions between personality and task context relate to differences in regional activation.

## Variation in medial prefrontal activation across contexts

### Medial prefrontal activity contexts influencing motivation and affect

Several research groups have manipulated task context and introduced affective content in order to investigate the effects on MPFC activation. Generally, manipulations aimed at influencing arousal and anxiety are associated with increases in MPFC activation, including additional neuronal generators in ventromedial regions. For example, Taylor et al. (2006) recorded fMRI responses while participants completed a modified flanker task involving blocks with different monetary incentives for performance. Their results show that, compared to the non-incentive condition, hemodynamic error responses in VMPFC regions were significantly greater when errors resulted in monetary loss. More recently, Simões-Franklin et al. (2010) employed a similar manipulation in a Go/Nogo task and found that phasic activation in the VMPFC (but not dorsal) to errors was significantly greater in the punishment compared to the neutral condition. In a gender voice decision task, involving neutral and emotional words and either congruent (i.e., auditory presentation and response side matching for gender) or incongruent (i.e., auditory presentation and response side not matching for gender) trials, Kanske and Kotz (2011) found additional recruitment of the VMPFC when participants resolved conflicting stimulus-response mappings, but only when the words were emotional. Together, these functional imaging studies illustrate that the engagement of the MPFC is sensitive to task context and the presence of affective content, and this is particularly the case for ventromedial regions. Similar results have been reported in several EEG studies.

Manipulating the monetary value of errors demonstrates that error-related brain responses are larger when mistakes result in punishment (Potts, 2011), or are associated with high compared to low monetary value (Hajcak et al., 2005). With respect to sources, VMPFC regions have been implicated when performance monitoring is being executed in arousing contexts, or when individuals are processing emotional information and feedback (Luu et al., 2003). For example, compared to verbal encouragement, derogatory feedback has been shown to increase ERN amplitudes (Wiswede et al., 2009) and, more recently, researchers have shown that verbal admonishment following erroneous responses is associated with additional recruitment of ventromedial sources (Ogawa et al., 2011), at least in females. The sex distribution of the sample may be important when examining the effects of arousing contexts of brain function. For example, in contrast to some of these results, Clayson et al. (2011) found that manipulating state affect had little influence on behavioral or ERP measures; however, forty percent of Clayson et al.'s (2011) sample (*n* = 69) was comprised of males, whereas the samples in the Wiswede et al. (2009; *n* = 28) and Ogawa et al. (2011; *n* = 15) studies were exclusively female.

Inhibitory N2s are also sensitive to arousal manipulations, as revealed by larger amplitudes (Potts, 2011) and ventral sources during conditions of distress and anxiety as compared to neutral and positive conditions (Lewis et al., 2006; Lamm et al., 2011). Overall, contextual demands influence functioning of the MPFC, and additional activation and recruitment of ventral sources of the MPFC may occur when tasks involve responding to emotional content, increased arousal, or motivational pressures.

### Medial prefrontal activity in social contexts

Other researchers have focused on the influence of social context on MPFC-related brain responses, introducing social pressures by including performance comparisons or by having participants monitor their performance in the presence of others. For example, Yu and Zhou (2006) found similar FRN effects (loss compared with gain), regardless of whether the feedback was self-relevant or related to another's performance. In other studies, increased FRNs were observed when feedback indicated that someone else had performed better (Boksem et al., 2011, 2012). Similar results were found when the research participant's outcome was yoked to that of another performer. Itagaki and Katayama (2008) collected FRNs in a gambling task to feedback which indicated whether or not the other's performance resulted in the participant winning or losing. Participants produced FRNs not only to the other person's losses, but also when the other person's wins resulted in losses for them. Marco-Pallarés et al. (2010) also found that FRN amplitudes were larger when outcomes resulted in wins for others and losses for the self, as compared to feedback indicating similar outcomes.

Using a social comparison model, Kim et al. (2011) had participants rate faces for attractiveness and then presented them with feedback about how deviant their rating was from an average. Medial frontal responses were found to be larger when feedback indicated that participants' responses differed from the group average.

These studies indicate that the response of the MPFC to evaluative feedback can be found in a wide variety of contexts, including those indicating subtlety of social comparisons. Considering the wide variation in personal responses to this kind of contextual information, these findings reinforce the need to consider individual differences in medial frontal responses.

## Variation in medial prefrontal activation across individuals

### Medial frontal negativities, personality, and temperament

We increasingly find studies focused on exploiting individual differences in MPFC activation, particularly with respect to variation in personality and temperament. In general, individuals who score higher on measures of behavioral inhibition, withdrawal, or negative affect produce larger medial frontal responses. Researchers working from Gray's (Gray, 1987, 1989) approach-avoidance model find that larger ERN amplitudes are associated with higher scores on the Behavioral Inhibition Scale (BIS) (Boksem et al., 2006a). Using a Go/Nogo task to collect MFNs, Amodio et al. (2008) reported that higher BIS scores were associated with larger amplitudes (i.e., more negative) of both the ERN and the N2. These effects remained after adjusting for scores on the Behavioral Activation System (BAS) and for the left-right frontal alpha asymmetry, suggesting that it is negative affect and not its associated withdrawal tendency that underlies the increased medial frontal activation in their study (Davidson and Irwin, 1999; Coan and Allen, 2003; Davidson, 2004). We have found similar effects in our lab when investigating medial frontal activation to monetary wins and losses in a gambling task. In line with others' results (Amodio et al., 2008), we showed that the level of punishment sensitivity correlated with FRN amplitude, even after accounting for reward sensitivity and sex differences. Although women demonstrated larger FRNs than men, the gender difference was accounted for by the women's higher levels of sensitivity to punishment. Consistent with the summary on sources outlined above, punishment sensitivity was also associated with greater activation in the VMPFC during the FRN (Santesso et al., 2011).

Similar to the focus on the approach-avoidance dimension, predispositions toward internalizing and externalizing in children relate to increased and decreased activation of the MPFC, respectively. Generally speaking, internalizing is characterized by maladaptive self-focusing on internal negative mood states (e.g., anxiety, depression), whereas externalizing reflects anti-social behavioral tendencies. Results from our lab show that in 10 year olds, poorer socialization (e.g., higher scores of lying and psychoticism) is correlated with smaller ERN amplitudes (Santesso et al., 2005). In a separate study, Stieben et al. (2007) reported that, compared to controls and those co-morbid for externalizing and internalizing tendencies, inhibitory N2 and ERN signals were attenuated in children with pure externalizing symptomatology. Similarly, Moadab et al.'s (2010) examined the N2 and ERN in 9–13 years olds using an emotional Go/Nogo task and found that these MFNs were larger in those scoring higher in internalizing.

Anxiety symptoms have also been related to VMPFC activation in terms of timing rather than amplitude. Lamm et al. (2011) used a Go/Nogo task involving a negative emotion induction (where the participant loses points) while obtaining Nogo N2 amplitudes in anxious aggressive 8–12 year-old children. During emotion induction, anxious aggressive children showed strong engagement of VMPFC regions during the early stages of inhibitory control (200–300 ms post Nogo stimulus), whereas non-anxious aggressive children showed the dominance of ventral regions during the later stages of behavioral inhibition (400–500 ms post Nogo stimulus). These patterns were interpreted as reflecting an early anxious response due to increased demands on cognitive control in the anxious-aggressive children, versus a later frustration response due to the increased pressure to regulate behavior in the non-anxious aggressive children.

Together, these results indicate the importance of medial prefrontal functioning and regional activation in temperament variation, and support how underlying mechanisms for these differences can be observed early in development.

### Medial frontal negativities and temperament factors in risk-taking

Investigating the neural correlates of approach-avoidance tendency is particularly relevant to understanding individual differences in risk-taking behaviors. Approach-related behaviors are core to risk-taking, and studies implicate deactivation of the MPFC during performance monitoring as a neural correlate of approach-related dispositions. In a sample of young males, Santesso and Segalowitz (2009) found that individuals scoring higher on sensation-seeking and reward sensitivity produced lower levels of medial frontal activity following erroneous behavioral responses. Similar effects were observed when we used a modified version of the Balloon Analogue Risk Task (BART) in a sample of 28 university students (van Noordt, 2012). In the standard BART, participants inflate a balloon in order to collect points or money, but are faced with the possibility that the balloon could pop, resulting in a loss of the accrued points. Risk-taking is indexed as the number of pumps on those trials on which the balloon did not pop, and is associated with approach-related behaviors (e.g., sensation seeking, impulsivity) and self-reported risk-taking (Lejuez et al., 2003b), as well as self-reports of addiction (Hopko et al., 2006) and detrimental health behaviors (Lejuez et al., 2002, 2003a). In our version, participants decided when to stop the continuous inflation of a balloon in order to collect their points, allowing us to record FRNs to loss feedback (i.e., trials in which the balloon popped which, in our task, also resulted in the participant losing 10% of their previously accumulated winnings). Using standardized low resolution brain electromagnetic tomography (sLORETA; Pascual-Marqui, 2002) to model source activation during the FRN, we found that CSD in the VMPFC correlated with risk-taking (i.e., the amount of time individuals permit the balloon to inflate on win trials), such that lesser VMPFC activation predicted a greater willingness to exhibit behaviors which ultimately become disadvantageous in the BART.

These effects are clarified further by research aimed at disentangling risk-taking profiles across contexts. Polezzi et al. (2010) found that FRN amplitudes did not differentiate between outcomes as long as participants were in their comfort zone for risk-taking (greater for some, less for others). In one condition the gains and losses were of equal magnitude (zero-expected value), whereas in another gains were larger than losses (positive-expected value). Individual brain responses to feedback did not differentiate between gains and losses only in the context in which the participant was more likely to take risks and seemed to be insensitive to the possibility of losing. Thus, individual differences in risk-taking behaviors relate to MFNs in terms of the subjective evaluation of risk.

### Medial frontal negativities and sociopolitical orientations

In addition to the associations described previously, activation of the MPFC has also been related to constructs seemingly more distal from biological temperament, including those reflecting social, political and religious orientations. In a sample of American undergraduates, Amodio et al. (2007) found that students who self-identified as being more liberal showed larger ERN and N2 amplitudes in a Go/Nogo task. We have recently replicated and extended these findings by showing that greater medial frontal activation is associated with a greater predilection for egalitarianism and social change and inversely with traditionalism (Weissflog et al., 2010). Similar to studies showing that conservative orientations relate to reduced engagement of the MPFC during performance monitoring, Inzlicht et al. (2009) found that stronger religious zeal and belief in god were associated with reduced electrocortical activation following error commission.

Given the dynamic developmental relations between activation and cortical growth, one might speculate that the relations should extend to tissue size as well. Unfortunately, there are few studies reporting actual physical size of the ACC and associated medial frontal structures as they relate to personality variables or social attitudes (although see, for example, Whittle et al., 2008a,b, 2009a,b) but one recent report is relevant to the social attitudes research described above. Kanai et al. (2011) reported that greater liberalism is associated with larger ACC size, and that greater conservatism is associated with increased size of the right amygdala. Such anatomical reports, if replicated, lead to intriguing hypotheses concerning how to characterize such differences in temperament, although they do not resolve the issue of cause and effect, considering the degree of plasticity of neural networking in both these structures and their sensitivity to experience (Vyas et al., 2002; Cook and Wellman, 2004; Mitra et al., 2005; Radley et al., 2006a,b; Dias-Ferreira et al., 2009; Liston et al., 2009).

### Medial frontal negativities and state-trait mood and affect: clinical samples

Both clinical and non-clinical levels of anxiety, neuroticism and emotionality relate to medial frontal activation. Generally, greater levels of anxiety (Goldin et al., 2009), worry (Endrass et al., 2010), neuroticism (Pailing and Segalowitz, 2004; Olvet and Hajcak, 2012), social distress (Eisenberger and Lieberman, 2004), negative affect (Luu et al., 2000; Olvet and Hajcak, 2012; Santesso et al., 2011), and emotional reactivity (Fukushima and Hiraki, 2009) are associated with increased activation of the MPFC.

Obsessive–compulsive disorder (OCD) is characterized by negative invasive thought patterns that engender anxiety and worry about subsequent remedial behaviors, and greater neural responses have been observed in clinical groups (Gehring et al., 2000; Ursu et al., 2003; Fitzgerald et al., 2005; Ruchsow et al., 2005; Endrass et al., 2008; Hajcak and Olvet, 2008) and in non-clinical samples with respect to obsessive-compulsive (OC) behaviors (Santesso et al., 2006), in children (Hajcak and Olvet, 2008) and adults (Endrass et al., 2008, 2010), and during both correct and error trials (Endrass et al., 2008). In addition to group-level effects, medial frontal activation has also been shown to increase as a function of symptom severity (Gehring et al., 2000; Xiao et al., 2011), with activation of ventromedial regions being especially related to symptomatology (Fitzgerald et al., 2005).

Similar to the findings in persons with OCD, hyperactivation of the performance monitoring system has also been observed in persons with depression (Chiu and Deldin, 2007; Holmes and Pizzagalli, 2008; Mies et al., 2011), including those in remitted stages of the disorder (Santesso et al., 2008; Georgiadi et al., 2011). In their study, Santesso et al. (2008) found that, compared to controls, persons with remitted depression had larger FRNs even after controlling for residual symptoms of anxiety and depression. Beyond group differences, higher levels of depressive symptoms are related to larger error-related brain responses (Fitzgerald et al., 2005; Chiu and Deldin, 2007; Weinberg et al., 2010), and the extent to which neural responses differentiate correct (or reward) from error (or loss/non-reward) responses is associated with depression severity (Foti and Hajcak, 2009; Olvet et al., 2010). However, some researchers have reported null or opposite effects (see Ruchsow et al., 2004, 2005; Schrijvers et al., 2008, 2009; Olvet et al., 2010).

Several reviews have focused on the functional significance of MFNs in relation to anxiety and performance monitoring (see Robinson et al., 2010; de Bruijn and Ullsperger, 2011; Lee and Park, 2011; Weinberg et al., 2012). Briefly, similar to the results from studies focusing on OCD or depression, individuals with high levels of generalized anxiety show larger electrocortical MPFC responses following errors (Weinberg et al., 2010; Xiao et al., 2011). Both ERN (Weinberg et al., 2010) and FRN (Gu et al., 2010) amplitudes have been shown to differentiate individuals' anxiety levels, such that more severe symptoms are associated with larger scalp negativities. These findings support the notion that errors provoke defensive responses, and that error-related brain responses may be a marker for individual differences in defensive reactivity (see Weinberg et al., 2012) and susceptibility to anxiety-related psychopathology (Olvet and Hajcak, 2008; Robinson et al., 2010).

The findings described above suggest that MFNs may represent a neurophysiological marker (i.e., endophenotype) for adaptive self-regulation of anxiety and arousal. However, one could also ask whether MFN amplitudes are a result of the person's psychological state and not a trait predisposition. This issue is, of course, difficult to resolve with human research participants because we cannot manipulate the clinical status or personality trait of the individual in order to see how this affects MFNs. It may be the case that a raised level of anxiety increases the reactivity of the medial frontal cortex, or it may be the case that a more reactive medial frontal cortex produces the anxiety symptoms. Evidence in favor of the latter position comes from the finding that persons with OCD produce similar ERNs regardless of punishment associated with their errors (Endrass et al., 2010), and that successful treatment does not attenuate ERN amplitude (Hajcak and Olvet, 2008). This suggests that MFNs may represent an endophenotype (Hajcak and Olvet, 2008; Ullsperger, 2009) of vulnerability for a limited capacity for adaptive self-regulation, and that when attenuation of symptoms result from treatment, this is not done by altering the underlying susceptibility of the person to the illness but by some top-down control over behavior and mental state. To definitively test this hypothesis, one would need to follow patients until complete remission is demonstrated, and then one might find a regularized MFN. However, such studies have not yet been done.

Such studies would be especially important for understanding the associations between brain function and symptomatology, given the evidence suggesting that MPFC functioning relates to treatment effects. For example, there is an extensive literature focused on the relationships between the structural and functional integrity of the VMPFC, particularly subgenual ACC [SGACC; Brodmann Area (BA) 25], and the regulation of mood and affect. Structurally, reduced gray matter volume in or near the SGACC has been found in persons with depression (Boes et al., 2008), in cases of early-onset depression (Botteron et al., 2002), as well as those suffering from other symptoms of mood dysregulation (Drevets et al., 2008). Functionally, the role of the SGACC in mood regulation is reflected in activation patterns. Individuals with family history of mood disorders have been found to exhibit reduced glucose metabolism in SGACC (Drevets et al., 1997), a finding which has also been reported in persons with depression characterized by anhedonia (Pizzgalli et al., 2004). The role of the SGACC, and more broadly the VMPFC, in regulating mood is supported by a growing body of evidence showing that dysregulation in fronto-limbic regions is associated with response to treatment.

In the 1990s, Mayberg and colleagues showed that individual differences in the activation of the cingulate cortex related to treatment efficacy, such that greater activation predicted better response to treatment (Mayberg, 1997; Mayberg et al., 1997). These findings have been extended using high-density EEG recordings in order to model CSD of theta power in the ACC (Pizzagalli et al., 2001). As is the case with hemodynamic measures, Pizzagalli et al. (2001) found that greater activation in multiple anatomical regions of the ACC prior to treatment predicted better outcomes post-treatment. Based on some of these findings, the SGACC has been proposed as an important cortical region which serves as a nexus for supporting processes of self-reference, as well as modulating the functional relationships between other prefrontal areas involved in cognitive control (see Pizzagalli, 2011, for a recent review). Using fMRI, the results from Yoshimura et al.'s (2010) study support the role of the VMPFC as an important cortical region involved in mediating emotional and cognitive self-control. These researchers report that cortical activation near the SGACC mediated the relationship between depressive symptoms and activation of other medial prefrontal regions involved in self-regulation. Taken together, there is good evidence that the functioning of the SGACC, and the VMPFC more generally, supports affective/evaluative processes and is associated with temperament, personality, and mood, especially in relation to negative affect and anxiety.

### Medial frontal negativities and state-trait mood and affect: non-clinical samples

In addition to clinical data, associations between cortical activation, personality, and mood are observed in sub/non-clinical samples (Xiao et al., 2011). Similar to those with clinical symptoms, college students who score high on the Obsessive Compulsive Inventory (Hajcak and Simons, 2002), as well as those scoring higher on measures of general anxiety (Hajcak et al., 2003; Xiao et al., 2011) or depression (Xiao et al., 2011), elicit larger ERNs than those scoring lower on these measures. The results of several studies show that factors such as fatigue (Boksem et al., 2006b) task involvement (Yeung et al., 2005; Tops and Boksem, 2010) and perceived responsibility for outcomes (Li et al., 2010, 2011) modulate MFN amplitudes. In addition, greater self-reported negative affect (Luu et al., 2000; Hajcak et al., 2004; Yasuda et al., 2004; Sato et al., 2005; Santesso et al., 2011) and neuroticism (Pailing and Segalowitz, 2004; Eisenberger et al., 2005; Olvet and Hajcak, 2012) also relate to enhanced neuronal activation to error or loss/negative feedback. Even more abstract constructs such as empathy have been found to relate to MFN amplitude, such that persons who are more empathic have larger (i.e., more negative) MFNs (Santesso and Segalowitz, 2009; Larson et al., 2010).

Anxiety in non-clinical samples dissociates physiological responses to error feedback. For example, Santesso et al. (2011) found that healthy adults with higher scores in negative emotionality produce larger FRNs to negative feedback in a monetary incentive task, as well greater activation in VMPFC, possibly reflecting rapid affective processing of negative feedback. In their study, Hajcak et al. (2004) found that, compared to those low in negative affect, individuals high in negative affect produced larger ERNs and greater skin conductance responses following errors. These findings suggest that higher levels of negative affect are associated with a systemic hyperactivation of the nervous system, as reflected by greater responses in both the central and autonomic branches. Similarly, with respect to the FRN, amplitudes have been shown to predict an individual's willingness to reject unfair offers. These decisions are associated with higher levels of negative affect and sympathetic activation (Hewig et al., 2011). Taken together, differences in temperament styles are reflected by the variability in MPFC activity between groups, as well as across individuals. Examining the associations among brain function, temperament, and personality is not only relevant to understanding the neural underpinnings of real-world behaviors, but can also be important for understanding dysfunctional cognitive and affective processes.

## Interactions between personality and context on medial frontal activation

Having summarized the effects of task demands and personality on activation of the medial frontal cortex, we should also examine interactions between these broad factors. Such interactions are critical for disentangling mediating and moderating factors in models of performance monitoring.

### Interactions in contexts involving performance-related incentives

It may be that the degree to which context affects brain responses varies in relation to personality characteristics. For example, we found that individuals who are high in conscientiousness are less sensitive to task manipulations aimed at increasing error significance, as reflected by their larger ERNs for all errors. Conversely, those lower in conscientiousness varied their ERNs as a function of how much their erroneous responses cost, showing larger ERNs when errors were associated with relatively more severe monetary punishments (Pailing and Segalowitz, 2004). Boksem and colleagues have also found interaction effects when investigating personality and temperament. Specifically, persons scoring high in behavioral inhibition not only generate larger ERNs (Boksem et al., 2006a), but this effect is also greater when errors are associated with losing money (Boksem et al., 2008). These data illustrate the interactions between context and personality on brain activation, given that persons who are behaviorally inhibited or have lower self-confidence are more sensitive to being punished for their mistakes, as reflected by their MPFC activity.

With respect to approach behaviors, extraverted individuals are considered to be more approach-oriented and driven by novelty, sensation-seeking, and rewarding outcomes (Campbell et al., 2003; Cohen et al., 2005). Smillie et al. (2010) manipulated feedback frequency (with 80% expected vs 20% unexpected) and outcome type (reward versus non-reward) and found that, compared to those scoring low on extraversion, individuals high in extraversion generated larger FRNs to unexpected reward outcomes, and smaller FRNs to unexpected non-reward outcomes. These results illustrate that those individuals who find novelty and rewards more salient have enhanced MPFC activation to unexpected reward and attenuated MPFC to non-reward outcomes, respectively. Together, these studies highlight how neither individual differences on traits related to performance monitoring nor task demands necessarily act on their own.

### Interactions in clinical samples

It is not surprising that interactions among context, personality and brain activation are observed in clinical samples. For example, we reported that incarcerated psychopaths produce attenuated error-related brain responses only when having to deal with affective stimuli that they are known to have difficulty processing (i.e., emotional faces). However, there was no difference in ERN amplitudes between psychopaths and controls when collected in response to errors on a standard letter flanker task (Munro et al., 2007a), suggesting that their performance monitoring system is as sensitive as that of controls when mistakes occur in a non-affective context. In addition, we found no evidence of inhibitory control deficiency in psychopaths, as indexed by N2 amplitudes in a non-affective paradigm, whereas non-psychopathic incarcerated offenders did produce attenuated N2 responses, possibly reflecting lower levels of inhibitory control (Munro et al., 2007b). These studies illustrate that the way the brain responds to performance feedback across contexts varies in relation to personality differences. Moreover, these results caution against treating all MFNs as reflecting a single construct considering that context can dissociate them.

Interactions between individual differences and context have also been investigated in other clinical samples characterized by mood dysregulation. The difference among the clinical presentations may be reflected in differences in the relative balance of regional activation across the MPFC. For example, symptoms of OCD, neuroticism, anxiety and negative affect may involve a relatively stronger engagement of the VMPFC as compared to dorsal regions (Fitzgerald et al., 2005). Support for this regional differentiation was reported by Gründler et al. (2009) and was explored further by Cavanagh et al. (2010), who found that individual differences in OC symptomatology were characterized by different MPFC activation profiles at rest and during performance monitoring. At rest, OC symptomatology correlated positively and negatively with activity in the VMPFC and DMPFC, respectively. Thus, even when there is no demand for performance monitoring, individuals more prone to experience negative intrusive thoughts and anxiety show an increased activity in medial prefrontal regions involved in saliency appraisal and sympathetic modulation (i.e., VMPFC). Moreover, these individuals show disengagement of regions typically recruited to when cognitive control is needed to regulate behavior (i.e., DMPFC). While monitoring their performance, individuals in the high OC group had hyperactivation of the VMPFC to errors on a flanker task and hypoactivation in the DMPFC to error feedback on a reinforcement learning task. These results suggest that when persons characterized by pathological levels of anxiety and worry make mistakes, they show larger responses in medial prefrontal regions implicated in feedback evaluation and affect regulation. Moreover, when these individuals fail to learn from feedback they produce relatively little activity in prefrontal regions involved in the cognitive control of behavior. Findings such as these not only illustrate the complexity of the interactions between brain activity, context, and individual differences, but also shed light onto how brain-behavior relationships may reflect maladaptive self-regulation.

### Interactions in non-clinical samples

Non-clinical experimental manipulations also reveal that personality and context interact to influence medial prefrontal responses. Olvet and Hajcak (2012) randomly assigned participants to be exposed to either neutral or sad media clips prior to performing a flanker task. They found that, following sad mood induction, greater self-reports of sadness were associated with larger ERNs. In addition, this effect was moderated by neuroticism such that persons higher in neuroticism exhibited a stronger coupling between sad mood and error-related brain responses. Using a different manipulation to punish errors on a flanker task, similar findings were reported by Riesel et al. (2012). In contrast to neutral blocks in which errors were never punished, 50% of errors were followed by an aversive sound in punishment blocks during the first half of the experiment (acquisition phase). Although the aversive sound to errors was removed during the second half of the experiment (extinction phase), participants still generated larger ERNs to these errors compared to errors made in neutral blocks. As would be predicted, the effect of punishment context on medial frontal activation was greater for persons scoring higher on trait anxiety. Thus, individuals who are more prone to worry and experience negative affect are especially sensitive to punishment-related contexts as reflected by electrocortical responses. Although these studies did not examine source activation, other studies focusing on these issues strengthen the association described earlier linking VMPFC regions with negative affect states in clinical populations and their effect on MFNs.

### Interactions in social contexts

**Figure 4:**
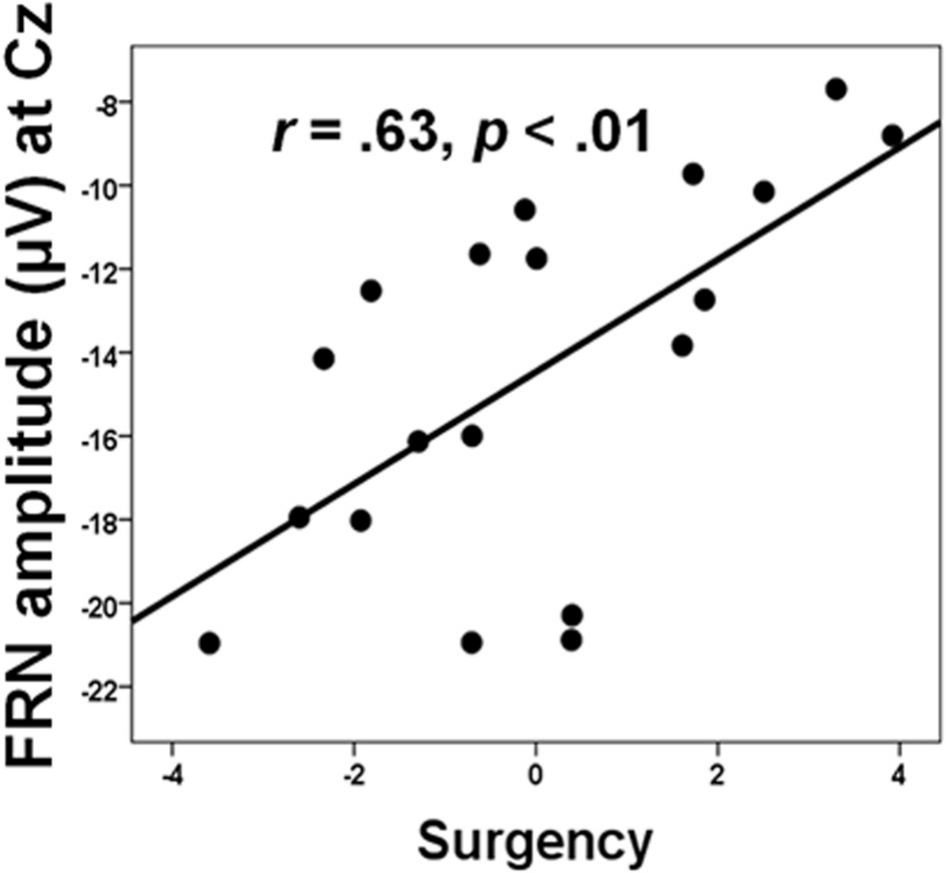
**The relation between FRN amplitude and surgency, a composite of BAS, sensation seeking (SSS-V), and positive affect (PANAS) in the “alone” condition.** Data from Segalowitz et al. (2012).

Social factors can also, of course, affect how individual differences in personality relate to medial frontal functioning, which has clinical as well as theoretical implications. From a clinical perspective, such effects may help identify which individuals have a predilection for maladaptive responses when their performance is worse than that of others, or when they are monitoring their performance in competitive situations. For example, Chein et al. (2011) showed that peer presence increased the activation of the incentive system (ventral striatum and orbitofrontal cortex) in adolescents when they were taking risks in a videogame designed to encourage dangerous driving. Peer presence did not influence adults in this way. In a similar research design, we found that peer presence selectively reduced the FRN produced by 15 year-old boys when they lost points due to excessive risk-taking in a similar videogame (Segalowitz et al., 2012). However, we also found that this effect was influenced by individual differences, where higher scores on sensation-seeking, behavioral activation, and sensitivity to reward (summed together as a measure of “surgency”) was associated with greater reduction in the FRN (see Figure 4). Regional source modeling especially implicated the VMPFC, although regions of the ACC, including those more dorsal, were also active. However, it is not possible from these data to discriminate between the possibilities that individuals higher in surgency engage in riskier behavior, particularly in the presence of their peers, as a result of hypoactivation in the VMPFC, or that the VMPFC activates less in these individuals because of their personality traits.

Focusing on the nature of the relationship among individuals, Newman-Norlund et al. (2009) examined the influence of friendship on performance monitoring. In their study, participants observed outcomes of virtual penalty kicks for characters labeled as stranger or friend. Even though the stranger-friend associations were established only during the experiment itself, observing a virtual friend's failure engaged performance monitoring networks to a greater extent than witnessing a stranger perform sub-optimally. These effects have been extended by Kang et al. (2010) who recorded FRNs while participants observed an actual friend or a stranger perform the Stroop task. Not only were FRNs larger for a friend's errors compared to those of a stranger, but this effect was mediated by the extent to which the participant considered their friend as part of their self-concept. Thus, watching a friend make mistakes engages performance monitoring networks to a greater degree than witnessing mistakes made by a stranger, and this engagement is larger when individuals perceive themselves to be closer to their friend.

A similar avenue of social neuroscience research focuses on the association between fairness of outcomes and medial frontal activation. In paradigms such as the Ultimatum Game, researchers have reported that highly unfair offers elicits greater MFN activation compared to more fair offers (Van der Veen and Sahibdin, 2011; Wu et al., 2011), and differentially impact peripheral nervous system responses, such as cardiac (Van der Veen and Sahibdin, 2011) and skin conductance responses (Hewig et al., 2011). In two recent studies, outcomes have been shown to interact with individuals' perceptions of fairness. Boksem and De Cremer (2009) collected FRNs to outcomes in the Ultimatum game and found that unfair offers were not only associated with larger amplitudes, but that the effect was strongest for individuals reporting high concerns for fairness. Using a different paradigm, the Dictator Game, Wu et al. (2011) found the FRN to be differentially sensitive to the fairness of outcomes depending on the source of the offer. Specifically, amplitudes were larger to unfair compared to fair outcomes when the offers were made by friends, whereas FRNs did not differ to when offers were made by a stranger.

### Interactions: genes, neurotransmitters, and personality

Some research has focused on the association between hormones (e.g., cortisol; Tops et al., 2006; Cavanagh and Allen, 2008; Tops and Boksem, 2011) and various neurotransmitters and performance monitoring processes. Several genetic polymorphisms have been shown to affect MFNs (see Jocham and Ullsperger, 2009; Ullsperger, 2009, 2011 for reviews). In the context of performance monitoring, levels of error-related brain activity and corrective behavior are a function of polymorphisms on the catechol-O-methyltransferase (COMT) genotype and, as a result, tonic levels of PFC dopamine (Mueller et al., 2011). Other researchers have focused on allelic differences in genes coding for prefrontal dopamine receptors which are also associated with variations in both error-related brain activity and post-error behavioral adjustments (Kramer et al., 2007).

Serotonin genes have also been associated with MFNs. The variant of 5-HTTLPR which has one or two repeats is associated with lower activity of the serotonergic system, whereas the homozygous long form allele is associated with increased functioning of the 5-HTT system. Fallgatter et al. (2004) found that individuals who have lower 5-HTT function (the short variant) elicit larger ERNs to errors on a letter flanker task. This finding fits well with studies showing that lower levels of serotonin levels are associated with higher levels of anxiety, negative emotionality, and depression (e.g., Karg et al., 2011), all of which are symptoms known to relate to hyperactivation in the MPFC. With respect to depression, Holmes et al. (2010) used fMRI to examine the association between tandem repeats on the 5-HTTLPR gene, medial frontal engagement, and performance on a flanker task. Their findings indicate that persons with low 5-HTT function (the short variant) not only have less conflict-related activation (incongruent correct – congruent correct) in the DMPFC, but also engage the VMPFC to a greater degree following errors (incongruent error – incongruent correct).

Thus, individuals who are more susceptible to mood dysregulation and psychopathology hyperactivate regions thought to be predominantly involved in the modulation of arousal and affect when they make mistakes (i.e., VMPFC). Furthermore, these individuals also show a relative disengagement of prefrontal regions involved in mediating cognitive control (i.e., DMPFC), specifically when there is an increase in the demand to regulate behavior. In addition to these elegant findings, long allele carriers were more accurate following errors, suggesting increased vigilance in performance monitoring after instances of failure in persons who have a higher functioning serotonergic system and are less likely to develop depression.

## Summary, conclusions and caveats

As we hope is evident from this review, factors affecting MPFC functioning and performance monitoring are indeed complex. The ERN, N2, and FRN are similar electrocortical responses generated by MPFC neurons, but are functionally distinct and reflect different aspects of performance monitoring. Similarly, although these MFNs have been localized to overlapping regional sources of the MPFC, distinct regions of the MPFC might differentially contribute to the generation of these ERP components. Due to these factors, MFNs, although having some similarities, should not be considered to reflect the same performance monitoring process. The complexity arises from the fact that these differences in brain function vary as a function of personality, task context, and their interactions.

Of course, although the interactions may be significant, caution should exercised when interpreting their complexity until replicated. Furthermore, there has been relatively little focus on the role of other cortical regions with respect to error and performance feedback processing despite consensus that we are seeking to understand the networks associated with performance monitoring, not the activation of single regions. This is not to say that research aimed at synthesizing our understanding of personality with the role of the MPFC in performance monitoring is unfruitful. On the contrary, the relationship between personality differences and MPFC function is symbiotic at a theoretical level in that individual differences in medial frontal responses can add to our understanding of personality constructs, yet individual differences in personality and temperament that relate to variability in MPFC activation may also provide us with important information concerning the nature of performance monitoring brain responses. In other words, knowledge about a personality construct such as neuroticism is aided by knowing its relation to the structure and functioning of specific MPFC regions, such as the magnitude of response or engagement of dorsal versus ventral MPFC and how the task demands alter these relationships. Similarly, our understanding of the MPFC is aided by seeing to which personality constructs its activation relates. In this sense, this research presents an iterative learning process that supports the formulation, testing, and interpretation of hypotheses focused on the associations between personality, context, and functioning of the MPFC. Note, however, that this iterative process implies a difficulty in attributing a single cause-effect relationship between function and structure. Rather, the MPFC structure may heavily influence how the person responds to the task, with clear implications for how we interpret their personality, yet their personality predispositions may also help shape the structure and functioning of their MPFC over time.

It is important to note that most of the research on individual differences and MPFC functioning rely on cross-sectional, correlational designs. A consequence of this type of research is that causation cannot be inferred from the data, nor does this research directly investigate the mechanisms driving the phenomena of interest. To repeat an example raised earlier, it is not possible to discriminate between the possibility that individuals higher in surgency engage in riskier behavior, particularly in the presence of their peers, as a result of hypoactivation in the VMPFC, from the possibility that the VMPFC activates less in these individuals because of their personality traits (Segalowitz et al., 2012). In addition, although regional source modeling especially implicated the VMPFC, other ventral and dorsal regions of the MPFC were also active. Thus, more sophisticated experimental designs and longitudinal data are needed in order to disentangle issues of cause and effect with respect to personality, task context, and functioning of the MPFC. These studies will be especially important for expanding our clinical understanding of personality and mood disorders, as well as the effectiveness of various treatments.

We should also keep in mind that a MFN represents more than single regional response. There is little debate that information processing in the brain relies on the dynamic coordination of multiple complex neural networks. In order to truly appreciate the neural bases of behavior, an understanding of how various brain networks coordinate their activities to support a given process will be crucial (Pourtois et al., 2010). Specifically, variability in the structural and functional connectivity between regions of the MPFC and subcortical structures might account for individual differences in personality and performance, as well as how these factors interact with task context to impact MFNs (e.g., Cohen, 2011). Another possible research avenue is using Independent Components Analysis (ICA; Makeig et al., 1996) to better isolate independent cortical processes that contribute to variability in performance monitoring and personality. Once identified, these functionally independent components can be source localized to better understand the regional dynamics underlying MFNs. Furthermore, considering how the activation of different independent components or sources varies over time is another way to gain insight about how individual differences in network functioning relate to personality and task context.

### Are MFNs reflecting a common generator and if not, does it matter?

Although the notion of the ERN, FRN and Nogo N2 reflecting a common source generator persists, we think it is clear that it must be the case that they have (at most) something in common and much distinctive. This is partly because the tasks that elicit them are different from each other in fundamental ways, and therefore something reflecting this difference must be coded in the brain signal. However, more importantly, the standard tasks that elicit these components differ in the degree of affect and arousal that they elicit, and there is much evidence that these factors are important. Such empirical support of the components having separate sources is easy to find: Not only do the measures not intercorrelate highly all the time, their variance sometimes maps onto behavior in different ways. For example as mentioned earlier, we found a dissociation between ERNs and the Nogo N2 within a group of violent offenders. Such dissociations indicate that the psychological variables driving at least some of the generator sources may differ for the various MFNs. However, to fully document such differences, studies need to include multiple MFN measures on the same participants, something rarely done. In addition, of course, as illustrated above in terms of LORETA analyzes, the actual regions responsible for the negativity measured at the scalp may differ considerably for the three components either in specific locations or, more likely, in the balance of contribution from the MPFC subregions. We suggest that the relative contribution of the cortical sources underlying the ERN, N2, and FRN may depend on the specific stimuli or context used and the degree of emotional arousal engendered by the task demands.

The use of MFNs as a reflection of MPFC functioning has become well accepted in the research community, a fact well documented by the growth in research literature involving these electrophysiological components. However, the issues raised in this review suggest that despite this relative acceptance, some of the basic assumptions needed for their interpretation remain to be verified by future research.

### Conflict of interest statement

The authors declare that the research was conducted in the absence of any commercial or financial relationships that could be construed as a potential conflict of interest.

## Footnotes

^1^Researchers vary on whether the MPFC includes all of, portions of, or none of the ACC (Sallet et al., 2011). For the sake of simplicity, we will consider the term MPFC to include the ACC in general, and will be more specific when needed. Several researchers offer a more detailed labelling of the cingulate cortex based on the results from structural (Vogt, 2009), functional (Shackman et al., 2011), connectivity (Beckmann et al., 2009) and receptor density distribution (Palomero-Gallagher et al., 2009) studies.

^2^An N2 component is generated in many contexts, not necessarily representing the same generator as in the Nogo context. When we refer to the N2, we are doing so only to the inhibitory nogo context within a Go-Nogo task.
